# Over-the-Counter Anti-inflammatory Supplements for Adjunctive Rheumatoid Arthritis Therapy: A Comprehensive Narrative Review

**DOI:** 10.14336/AD.2024.0131

**Published:** 2024-01-31

**Authors:** Saliba Fares, Mourad Omar, Aoun Laurence, Saif Abu-Baker, Almardini Shaza, Haddadin Fadi, Mina Jonathan, Khattar Georges, Sangaraju Koushik, Bou Sanayeh Elie, Morcos Zeina, Habib Toni, Al Saidi Ibrahim, Slobodnick Anastasia

**Affiliations:** Department of Internal Medicine, Staten Island University Hospital, Staten Island, New York, USA.

**Keywords:** chronic disease, rheumatoid arthritis, dietary supplements, anti-inflammatory agents, oxidative stress, complementary therapies, biological products

## Abstract

Rheumatoid arthritis (RA), a prevalent chronic disease, poses significant treatment challenges, including side effects and high costs of conventional therapies. This heightens interest in natural, over-the-counter (OTC) anti-inflammatory supplements as potential adjunctive treatments. Given their roles in mitigating inflammation and oxidative stress, key factors in RA pathogenesis, these supplements could offer valuable therapeutic adjunctive. Our review addresses this emerging area, providing insights into the efficacy and safety of these supplements in RA management. Methods: observational studies, systematic reviews, and meta-analyses on anti-inflammatory supplements in RA over the past 10 years. Relevant articles were reviewed, and data was synthesized. Several supplements demonstrate beneficial anti-inflammatory and antioxidant properties that may improve RA outcomes. Quercetin suppresses inflammatory cytokines and pathways. Green tea polyphenols inhibit mediators like TNF-alpha and IL-6. Curcumin impacts inflammatory cytokines and signaling cascades. Ginger components block enzymes and inflammation pathways. Omega-3 fatty acids modulate immune cell function and cytokine production. While promising, high-quality evidence remains limited, and optimal dosing is uncertain for most supplements. Our review emphasizes the potential of over-the-counter anti-inflammatory supplements as an adjunctive treatment in rheumatoid arthritis. While these supplements, like quercetin, green tea polyphenols, and omega-3 fatty acids, show promising anti-inflammatory and immunomodulatory effects, there are substantial concerns regarding their consistency and quality in the market, unclear interactions with conventional medications, and the lack of standardized dosages. More extensive research, particularly randomized controlled trials, is crucial to establish clear guidelines.

## Introduction

Chronic diseases have become an increasingly prevalent concern in the global health landscape, affecting millions of individuals and posing significant challenges to both patients and healthcare providers. In a study published by the CDC, more than half (51.8%) of adults had at least 1 of 10 selected diagnosed chronic conditions (arthritis, cancer, chronic obstructive pulmonary disease, coronary heart disease, current asthma, diabetes, hepatitis, hypertension, stroke, and weak or failing kidneys), and 27.2% of US adults had multiple chronic conditions [1]. The prevalence of chronic diseases, ranging from cardiovascular disorders to autoimmune conditions, has seen a notable surge in recent years [2].

Among these conditions, Rheumatoid Arthritis (RA) emerges as a significant player. This chronic inflammatory condition impacts the joints, causing damage to cartilage and bone. This complex disorder is associated with significant mortality and morbidity. The worldwide prevalence is approximately 1%, with a higher incidence among women compared to men. It commonly affects people between the ages of 40 to 50 [3] and the clinical manifestations in its early stages may not always be apparent. Globally, musculoskeletal conditions, including RA, accounted for 18.5% of years lived with disability in 2015, marking a 68% rise from the figures recorded in 1990 [4]. Both genetic and environmental factors contribute to the development of RA. Risk factors include age, body weight, gender, family history, and smoking habits. Predominant genetic risk factors comprise HLA and non-HLA genes [5]. RA is characterized by three main features: inflammation of the joints and surrounding tissues, deterioration of cartilage, and erosion of bone [6]. The prevalence of RA not only underscores its societal impact but also emphasizes the necessity for innovative and multifaceted approaches to its management.

Early diagnosis and treatment are critical for optimal outcomes in RA, since high disease activity, presence of autoantibodies, and early joint damage are linked to poorer prognosis. [7] The therapeutic process entails assessing disease activity through comprehensive evaluation scores, pursuing a "treat-to-target" approach, and employing both established and innovative medications designed to alter the course of the disease. Once the treatment objectives of either remission or minimal disease activity are attained, adjustments can be made to reduce dosage or extend treatment intervals [8]. Medical treatment options often involve disease-modifying anti-rheumatic drugs to suppress inflammation. Although there have been advancements in patient outcomes due to the introduction of novel therapies, not all individuals respond positively to current treatments, underscoring the ongoing demand for innovative therapeutic options [9].

Throughout history, natural remedies have shown promise as potential treatments for RA. Considering the notable side effects associated with conventional treatments for RA, especially with prolonged use, patients are increasingly showing interest in over the counter (OTC) supplements as complementary options to standard therapy [10]. According to research conducted by the World Health Organization (WHO), 80% of individuals with RA have turned to traditional remedies and dietary modifications. These interventions encompass herbal remedies, phytochemicals, as well as dietary and nutritional elements [11]. It is imperative for healthcare professionals to possess evidence-based knowledge regarding these RA supplements to offer appropriate guidance to their patients.

Supplements available without a prescription, such as fish oil, vitamin D, Curcumin, and various others, are among the most frequently employed complementary and alternative treatments for promoting overall human health in the United States. Addressing musculoskeletal issues represents the primary motivation for their use [12]. While the overall utilization of these OTC supplements among adults has exhibited stability over the past decade, there have been shifts in the specific products selected, as evidenced by sales data and national health surveys [10]. For example, there has been a notable rise in the utilization of fish oil, turmeric, and ginger, whereas the usage of glucosamine and/or chondroitin sulfate has experienced a decline. Survey data indicates a heightened prevalence of OTC supplement usage among women, individuals taking prescription medications, and those grappling with chronic medical conditions [13]. Hence, it comes as no surprise that the consumption of these supplements is even more prevalent among adults with RA [14]. Although there are documented reports of side effects and potential interactions between OTC supplements and medications, there is a noticeable gap in studies concerning interactions specifically with drugs employed in the treatment of RA [15].

The role of complementary medicine in RA has been explored in previous studies [16-198]. Our review stands out for comprehensively examining a diverse array of supplements, going beyond a singular focus, and encompassing supplements with distinct mechanisms of action. Moreover, we provide analysis of studied doses, offering insights into the optimal levels for therapeutic efficacy. This review contributes to the growing body of knowledge in the field and provides evidence-based data for both researchers and practitioners seeking a comprehensive understanding of supplement-based approaches to RA management.

## Methodology

A comprehensive literature search was conducted in PubMed, EMBASE, and Google Scholar databases for articles published over the past 10 years on the use of over-the-counter anti-inflammatory supplements in rheumatoid arthritis. The following keywords were used in various combinations: rheumatoid arthritis, osteoarthritis, supplements, over the counter, anti-inflammatory, curcumin, turmeric, ginger, omega-3 fatty acids, fish oil, green tea, polyphenols, vitamin D, willow bark, quercetin. Additional pertinent studies were identified by manually screening the reference lists of retrieved articles. We included clinical trials, observational studies, systematic reviews, and meta-analyses focusing on the anti-inflammatory effects, efficacy, safety, mechanisms of action, and dosage recommendations for the supplements in rheumatoid arthritis patients. Studies conducted only in animal models or cell cultures were excluded. Relevant data from eligible studies were extracted and synthesized to provide an overview of the current evidence on these supplements as potential adjunctive therapies in rheumatoid arthritis.

## Pathophysiology

RA development results from intricate interactions between genetic susceptibility and environmental factors. Although the exact causes remain uncertain, it is believed that environmental triggers, such as cigarette smoke, can stimulate innate immune responses in mucosal sites such as the lungs. This stimulation leads to the activation of enzymes called peptidyl arginine deiminases (PADs), which modify proteins by converting arginine to citrulline. These altered proteins are then presented to T cells, leading to the production of antibodies against them, such as anti-citrullinated protein antibodies (ACPAs). ACPAs and inflammatory cytokines gradually increase years before the onset of RA symptoms. Another trigger, such as immune complexes, may be necessary to initiate joint inflammation. This permits the entry of autoantibodies, cytokines, and other inflammatory mediators that contribute to synovitis. The inflamed synovium becomes highly invasive, causing damage to cartilage and bone. Early intervention is crucial to prevent irreversible joint damage. The relative importance of underlying factors changes over time and varies among patients. Genetics and environmental factors set the stage for the disease, while antibodies and cytokines mark the pre-clinical phases. Local joint processes like fibroblast activation, immune complexes, and increased vascular permeability then led to clinical arthritis [19].

T cells react to modified self-antigens, stimulate autoantibodies, differentiate into inflammatory subsets, and drive chronic activation via co-stimulation. B cells propagate inflammation through autoantibody production, antigen presentation, T cell help, and cytokine release. Macrophages critically drive synovitis and damage through recruitment, activation, and release of inflammatory mediators. Aggressive fibroblast-like synoviocytes invade cartilage by secreting collagenases like MMP-13. Complex cytokine networks including TNF, IL-6, IL-17, and induction of GM-CSF initiate and perpetuate all facets of rheumatoid pathology. Key immune cells and their products represent major therapeutic targets in RA [19].

## Quercetin

Quercetin, a naturally occurring flavonoid compound present in various fruits, vegetables, and grains, has displayed potential advantages in the treatment of RA due to its anti-inflammatory and antioxidant properties when used as a supplement. Preliminary research has indicated that quercetin can reduce inflammation by lowering proinflammatory cytokines, inhibiting the production of reactive oxygen species, balancing immune cell activity, and restraining the invasion of fibroblast-like synoviocytes and osteoclastogenesis (Table 1).

**Table 1:** Animal studies and Clinical trials on Quercetin.

First Author	Year and Country	Dose	Participants	Measured Factors	Outcome
Choi et al. [22]	2009 Korea	0.5% quercetin or 0.05% vitamin E for 9 weeks	DBA/1J mice with collagen-induced arthritis	TNFα, IL-1β mRNA expression, MCP-1, PGE2, Nitric Oxide	VE diet increased inflammatory cytokines and markers. Quercetin and vitamin E suppressed inflammation; quercetin was more effective.
Javadi et al. [23]	2016 Iran	500 mg/day quercetin for 8 weeks	20 women with RA, aged 19-70 years	Hs-TNFα, EMS, TJC & SJC, DAS-28, HAQ	Reduced morning pain, after-activity pain, EMS, hs-TNFα level, DAS-28 and HAQ scores. Improved well-being. No significant effect on ESR and SJC.
Kai Yuan et al. [21]	2020 China	Not reported	Adjuvant-induced arthritis (AA) model in mice	IFN-γ, TNF, IL-6, arthritis scores, neutrophil infiltration and apoptosis, NET	Quercetin reduced ankle swelling, arthritis scores, neutrophil infiltration, inflammatory cytokine levels. Increased apoptosis in activated neutrophils. Inhibited NET formation by suppressing autophagy.

### Mechanism of action

Quercetin's primary mechanisms of action in RA involve the regulation of pathways associated with inflammation and oxidative stress. In experiments conducted in rats, quercetin has demonstrated the ability to reduce the production of inflammatory cytokines such as TNF-α, IL-1β, IL-6, and IL-8. This effect is thought to be achieved by blocking intracellular signaling pathways such as MAPK and NF-kB. Moreover, quercetin enhances the body's antioxidant defenses by influencing Nrf2 and inducing HO-1 [20]. Other proposed mechanisms include modifying the differentiation of immune cells, restraining the proliferation and invasion of fibroblast-like synoviocytes, and inhibiting osteoclast formation and matrix metalloproteinases. These multifaceted cellular actions collectively contribute to a decrease in joint inflammation, pain, and structural damage in animal models of RA [21].

### Clinical trials

A study by Choi et al. utilizing a mouse model of collagen-induced arthritis (CIA) demonstrated reduced cytokine and serum inflammatory markers among mice receiving a vitamin E-depleted diet, but not those receiving a normal diet.

Despite the impact on inflammation, there were no significant differences in clinical scores or joint histology between the groups, which might be attributed to the relatively short duration of the study. This trial suggests that antioxidant deficiency could worsen inflammation in RA, while supplementation with quercetin has the potential to mitigate inflammatory responses [22].

A double-blind, placebo-controlled randomized trial conducted by Javadi et al. examined the impact of quercetin supplementation on 50 women diagnosed with RA. The patients were randomly allocated to receive either 500mg/day of quercetin or placebo capsules in addition to their regular RA treatment for a period of 8 weeks. The study assessed various outcomes, including plasma levels of TNF-alpha, ESR (erythrocyte sedimentation rate), morning stiffness, pain, counts of tender and swollen joints, the disease activity score (DAS-28], physician global assessment, and the health assessment questionnaire (HAQ) disability index. The findings revealed that quercetin supplementation led to a significant improvement in morning stiffness, pain, DAS-28, HAQ scores, tender joint count, and TNF-alpha levels and a non-significant difference in ESR, swollen joint count and physician global assessment when compared to the placebo group and the baseline measurements [23].

### Recommended dosage

Based on current evidence, daily doses of 500-1000 mg quercetin appear safe and potentially effective for RA [23]. Maximum plasma concentrations are achieved around 2 hours after ingestion. Longer term human trials are still required to confirm benefits and optimal dosing [24].

## Green tea polyphenols

Green tea, originating from the Camellia sinensis plant, has a rich history of consumption in Asian nations including China and Japan. It is enriched with polyphenolic compounds, including epigallocatechin gallate (EGCG), which possess antioxidant and anti-inflammatory properties. Researchers have explored the potential advantages of green tea extracts and supplements in addressing inflammatory disorders such as RA (Table 2).

**Table 2:** Animal studies and clinical trials on green tea polyphenols.

First Author	Year and Country	Dose	Participants	Measured Factor	Results
Singh et al. [25]	2003 USA	100μM EGCG	OA chondrocytes from 7 patients	Effect of EGCG on IL-1β induced JNK activation	EGCG inhibited IL-1β induced JNK (especially p46 isoform) activation and phosphorylation of c-Jun. No effect on p38 or ERK.
Haqqi et al. [28].	2005 USA	0.2% GTP water solution	Male DBA/1 mice	Effect of GTP on collagen-induced arthritis	GTP reduced incidence and severity of arthritis. Lower inflammation markers and joint damage.
Lin et al. [29]	2008 Taiwan	20 mg/kg EGCG	Male Lewis rats	Effect of EGCG on collagen-induced arthritis	EGCG alleviated severity of CIA, reduced CCL2 expression and macrophage infiltration in joints.
Morinobu et al. [30]	2012 Japan	20 μg/gm body weight EGCG	Male DBA/1 mice	Effect of EGCG on antibody-induced arthritis	EGCG suppressed osteoclast differentiation in vitro. Ameliorated arthritis symptoms and joint damage in vivo.

### Mechanism of action

The polyphenolic compounds found in green tea, particularly EGCG, appear to offer various mechanisms that could potentially be advantageous for individuals with RA. EGCG has been documented in research studies as having the ability to inhibit inflammatory mediators such as tumor necrosis factor-alpha (TNF-α), interleukin-1beta (IL-1β), interleukin-6 (IL-6), and interleukin-8 (IL-8) [25]. Additionally, it appears to suppress inflammatory signaling pathways including nuclear factor kappa B (NF-kB) [26]. Moreover, EGCG exhibits antioxidant properties by neutralizing reactive oxygen species that contribute to inflammation [27]. Through these means, green tea polyphenols may have the potential to diminish inflammation and mitigate joint damage in RA.

### Clinical studies

The potential disease-modifying impact of EGCG on arthritis was initially revealed in a study where the inclusion of EGCG-containing green tea extract (GTE] in the drinking water improved CIA in mice [28]. This improvement in CIA incidence and severity was evident in the significant suppression of inflammatory mediators such as COX-2, IFN-γ, and TNF-α in the arthritic joints of mice that consumed green tea [28]. Furthermore, lower levels of total immunoglobulins (IgG) and type II collagen-specific IgG were observed in the serum and arthritic joints of the green tea-fed mice [28].

Interestingly, recent pharmacological investigations employing EGCG or green tea to mitigate arthritis have equally emphasized the reduction of bone resorption seen in RA [29]. A recent study by Morinobu et al. demonstrated that EGCG treatment reduced bone resorption as indicated by the presence of tartrate-resistant acid phosphatase-positive multinucleated cells, decreased bone resorption activity, and altered the expression of osteoblast-specific genes, particularly the transcription factor NF-ATc1 [30]. Another study employing a mouse CIA model revealed that EGCG (administered intraperitoneally daily at 20 mg/kg) ameliorated arthritis and macrophage infiltration while reducing the number of osteoblasts synthesizing MCP-1/CCL2 [29]. There has been no testing of the effectiveness of EGCG in human patients with RA through controlled phase trials.

### Recommended Dosage:

According to the latest research findings, the advisable dosage for green tea polyphenols such as EGCG is typically 500mg, either once or twice a day. It's important to note that exceeding 800mg may elevate the risk of liver toxicity, so it is recommended to limit dosages to under 800mg per day [31]. For optimal results, green tea extracts standardized to contain 70-80% polyphenols are preferred. To enhance absorption, it is advisable to take green tea supplements with a meal [31]. To sum up, a suitable supplemental dosage for individuals with RA ranges from 500mg to 1000mg per day of standardized green tea extract, divided into one or two doses.

## Vitamin D

Vitamin D is an essential micronutrient obtained through sunlight exposure, diet, and supplements. While vitamin D has long been recognized for its role in bone health, research now indicates it also serves as an influential regulator of immune function [32] This immunomodulatory capacity has implications for RA. Studies suggest vitamin D may temper aspects of the immune response involved in RA. Specifically, vitamin D appears capable of modulating T cell populations, including suppression of proinflammatory Th17 cells that drive RA pathogenesis [33]. Through these immunologic mechanisms, vitamin D supplementation is being investigated as a potential therapeutic adjunct in the management of RA (Table 3).

### Mechanism of action:

The biologically active form of vitamin D, known as 1,25-dihydroxyvitamin D3, can attach to vitamin D receptors present on various immune cells, including T lymphocytes, antigen-presenting cells, and monocytes/macrophages. This interaction initiates changes in gene expression that decreased pro-inflammatory cytokines such as IL-17 and TNF-alpha, while increasing anti-inflammatory cytokines such as IL-1029. [34] By inhibiting these cells and fostering immune tolerance, vitamin D potentially mitigates the inflammatory processes central to RA. Additionally, vitamin D might influence the activation of toll-like receptors and the maturation of dendritic cells, further restraining inflammatory immune responses [35].

### Clinical studies:

Multiple RCTs have been conducted to assess the effectiveness of vitamin D supplementation in patients with RA. These studies have employed a range of dosing strategies, varying from weekly doses of 50,000 IU to large, intermittent bolus doses ranging from 300,000 to 500,000 IU [36]. Another study in 2017, led by Chandrashekara et al., involved giving 60,000 IU of vitamin D weekly for six weeks, followed by monthly doses for 12 weeks to 150 RA patients. This open-label study reported notable improvements in DAS28 scores and vitamin D levels following supplementation. [37]. Additionally, some trials have focused on the impact of vitamin D on inflammatory markers. For instance, a 2017 RCT by Buondonno et al. investigated immune factors and vitamin D in early rheumatoid arthritis (eRA). The study evaluated adding high-dose vitamin D to standard methotrexate and glucocorticoid treatment in eRA patients. Adding vitamin D, significantly improved overall health after 3 months compared to standard treatment alone. [38] Additionally, a study by Gopinath in 2011assessed adding vitamin D to triple disease-modifying antirheumatic drug (DMARD) therapy for pain relief in eRA. 59 patients received 500IU vitamin D3 + calcium along with triple DMARDs, while 62 controls received triple DMARDs + calcium. After 3 months, 50% of the vitamin D group achieved pain relief versus 30% of controls using the visual analogue scale (VAS) for pain assessment. 68% of RA patients were vitamin D deficient, comparable to typical prevalence for vitamin D deficiency for the population which was studied. Vitamin D levels did not correlate with disease activity. [39]

**Table 3:** Animal studies and clinical trials on Vitamin D.

First Author	Year and Country	Dosage	Participants	Measured Factor	Results
Chang et al. [34]	2010 USA	50 ng 1,25D3	Mice	Effect of 1,25D3 on EAE	1,25D3 protected mice from EAE, reduced IL-17 and IL-17F production. Induced CHOP and inhibited Th17 cytokine production.
Széles et al. [35]	2009 Hungary	10 nM 1,25D3	Human monocyte-derived DCs	Effect of 1,25D3 on gene expression	1,25D3 regulated immunity/defense genes independently of differentiation. Autonomous regulation of tolerogenic phenotype.
Chandrashekara et al. [37]	2015 India	60,000 IU vitamin D per week	149 RA patients	Effect of vitamin D on RA disease activity	Vitamin D deficiency associated with active RA. Supplementation improved disease activity in vitamin D deficient patients.
Buondonno et al. [38]	2017 Italy	300,000 IU cholecalciferol	39 early RA patients	Effect of vitamin D on early RA	Vitamin D improved global health but did not affect T cell subsets or osteoclast precursors.
Gopinath et al. [39]	2011 India	500 IU 1,25D3 + calcium	121 early RA patients	Effect of 1,25D3 on early RA pain	1,25D3 significantly improved pain relief compared to calcium alone in early RA.
Franco et al. [40]	2018 Brazil	Various vitamin D dosages	640 RA, 505 SLE, 6 SSc patients	Effect of vitamin D on rheumatic disease activity	Vitamin D reduced anti-dsDNA positivity in SLE and may reduce RA recurrence, but more studies needed.

Despite these individual studies, overall findings remain mixed. A meta-analysis by Franco et al. concluded there is no definitive evidence supporting the clinical benefits of vitamin D supplements on measures of pain (visual analog scale] or disease activity in RA. Vitamin D supplementation was also associated with a non-significant decrease in RA recurrence. [40] This diversity in research outcomes underscores the necessity for more extensive, high-quality RCTs focusing on specific RA patient groups to determine the consistent therapeutic value of vitamin D. As it stands, the evidence regarding the clinical efficacy of vitamin D supplements as an adjunct treatment in RA remains inconclusive.

### Recommended dosage:

At present, standardized dosing guidelines for vitamin D specifically tailored for RA are not established. General suggestions range from 600 to 4000 IU per day for immune system modulation, with a cautionary note to monitor for possible toxicity in cases where high doses are administered over a prolonged period [41]. For RA patients exhibiting vitamin D deficiency, some research indicates that daily doses between 1000 and 4000 IU could offer anti-inflammatory benefits and potentially modify the disease course [42]. Nonetheless, the determination of optimal target levels and supplementation strategies for vitamin D in RA remains an area that requires more conclusive research.

## Curcumin

Curcumin, a polyphenol extracted from the Curcuma longa plant, commonly known as turmeric, has a rich history in Asian countries as both a culinary spice and traditional medicine, valued for its anti-inflammatory and antioxidant properties. This has led to its popularity in Western countries as a nutritional supplement, with a growing body of scientific evidence supporting its potential health benefits. Curcumin supplements are now widely available, primarily marketed for managing inflammatory conditions such as RA [43] (Table 4).

### Mechanism of action

Curcumin's capacity to mitigate inflammation and modulate immune responses stems from its interaction with various molecular components involved in inflammatory processes. It has been demonstrated to inhibit crucial inflammatory cytokines such as TNF-α, IL-1, and IL-6, as well as to impact signaling pathways such as NF-kB and JNK [44]. Additionally, curcumin hampers the activation and differentiation of inflammatory immune cells, including macrophages, dendritic cells, and T cells. These actions collectively contribute to curcumin's strong anti-inflammatory properties.

**Table 4:** Animal studies and clinical trials on Curcumin.

First Author	Year and Country	Dosage	Participants	Measured factors	Results
Fu et al. [44]	2016, China	Curcumin 12.5, 25, 50 mg/kg	Female lactating BALB/c mice	Mastitis inflammation and markers, TLR4/NF-κB signaling	Curcumin reduced inflammation, MPO, cytokines, TLR4 expression and NF-κB activation in LPS-induced mastitis model.
Chandran et al. [45]	2012, India	Curcumin 500mg, diclofenac sodium 50mg	45 RA patients	DAS28, ACR response	Curcumin improved DAS28 and ACR scores compared to diclofenac in RA, with no adverse events.
Pourhabibi-Zarandi et al. [46]	2022, Iran	Curcumin 500mg	48 RA patients	Metabolic factors, inflammation, adiposity	Curcumin improved TG, weight, BMI and waist circumference when compared to placebo
Javadi et al. [47]	2019, Iran	Curcumin nanomicelle 40mg	65 RA patients	DAS28, TJC, SJC	Curcumin nanomicelle improved DAS28, TJC and SJC from baseline, but not significantly different from placebo.

### Clinical trials:

In a randomized controlled trial conducted by Chandran and Goel in 2012, forty-five RA patients were divided into three groups to receive daily doses of either 500 mg of curcumin, 50 mg of diclofenac sodium, or a combination of both for 8 weeks. All treatment groups exhibited significant improvements in their DAS28 scores, but the curcumin group demonstrated the most notable improvement in both DAS28 and ACR scores (ACR 20, 50, and 70), outperforming the diclofenac sodium group. Crucially, curcumin treatment was deemed safe, without any associated adverse events, marking the first instance of evidence supporting curcumin's safety and effectiveness over diclofenac sodium in patients with active RA [45].

In a double-blind, placebo-controlled study led by Fatemeh Pourhabibi-Zarandi Et al., the impact of curcumin supplementation on metabolic indicators, inflammation, and obesity metrics was assessed in 48 women with RA. Participants received either 500 mg/day of curcumin or a placebo for 8 weeks. The study found that curcumin supplementation notably reduced several parameters: homeostatic model assessment for insulin resistance (HOMA-IR), erythrocyte sedimentation rate, serum high-sensitivity C-reactive protein, triglycerides, body weight, body mass index (BMI), and waist circumference, in comparison to the placebo. Conversely, the placebo group saw significant increases in HOMA-IR and triglycerides. No substantial changes were observed in fasting blood sugar, insulin, other lipid profiles, or visfatin levels. These findings suggest that incorporating curcumin into treatment plans could effectively enhance metabolic factors, reduce inflammation, and address obesity-related issues in patients with RA. [46]

In a 12-week double-blind trial, Maryam Javadi and colleagues investigated the effects of curcumin nano-micelle supplementation on 65 patients with RA. The patients were randomly assigned to either a group receiving 40 mg of curcumin nano-micelle three times daily or a placebo group. At the study's outset and conclusion, there were no significant differences between the groups in Disease Activity Score (DAS-28), tender joint count (TJC), or swollen joint count (SJC). However, both groups experienced significant reductions in DAS-28, TJC, and SJC from their initial levels, with the curcumin group showing slightly greater, though not statistically significant, reductions. No notable changes in erythrocyte sedimentation rate (ESR) were observed between the groups. The study indicates that adding curcumin nano-micelle to treatment may yield modest improvements in disease activity scores and joint symptoms in RA, suggesting its potential usefulness [47].

### Recommended dosage:

Current clinical research, though limited, has utilized a curcumin dose of 500 mg daily in patients with RA [45] [46]. However, the most effective therapeutic dose remains uncertain due to the diverse formulations used in studies and the absence of comparative analyses across different dosages. While doses as high as 8 grams per day have been deemed safe and tolerable, there's no concrete evidence suggesting that higher doses yield enhanced benefits [48]. Curcumin supplements should be consumed with meals and combined with piperine, which aids in increasing their bioavailability. To establish the most effective dosage of curcumin for alleviating symptoms of RA, more extensive dose-response studies are necessary.

## Ginger

Ginger, the familiar spice that adds zing to our culinary creations, is increasingly emerging as more than just a flavour enhancer. Scientifically termed *Zingiber officinale Roscoe*, this humble plant is proving to be a potential ally in the management of RA [49]. Belonging to the *Zingiberaceae* family and boasting over 300 varieties, ginger carries a host of health benefits, from helping with nausea to easing osteoarthritis pain and even contributing to glycemic control. [50] Ginger has also become a subject of interest in the context of RA management (Table 5).

**Table 5:** Animal studies and clinical trials on Ginger.

First Author	Year and Country	Dosage Used	Participants	Measured Factor	Results
Naheed Aryaeian et al. [62]	2019, Iran	1.5 grams of ginger powder daily	66 patients with RA, aged 19-69 years	Ginger's effect on cytokines gene expression in RA	Significant decline in CRP and IL-1β mRNA level; reduced TNFα mRNA levels (not significantly); no effect on IL2 gene expression
Søren Ribel-Madsen et al. [54]	2012 Danmark	100μg/mL of standardized ginger extract	Synoviocyte cell cultures from OA, RA and healthy controls	Cytokine production (IL-1β, IL-6, IL-8, etc.)	Ginger extract reduced cytokines similarly to betamethasone, ibuprofen had little effect.
Sudipta Tripathi et al. [57]	2008 USA	1 μl/ml alcoholic ginger extract	Murine peritoneal macrophages	Cytokine (TNFα, IL-12, IL-1β), chemokine (RANTES, MCP-1) production, surface marker expression (B7.1, B7.2, CD40, MHC-II)	Ginger extract decreased cytokine and chemokine production, APC function and T cell activation/proliferation
Richard B. van Breemen et al. [59]	2011 USA	N/A	N/A	Binding to COX enzymes by ginger compounds (e.g. 10-gingerol, 8-shogaol), inhibition of COX by ginger extract and compounds	Multiple ginger compounds bound COX-2 and inhibited enzyme activity, with selectivity over COX-1. 10-shogaol most potent inhibitor.

### Mechanism of Action:

At the heart of ginger's therapeutic potential lie its bioactive compounds such as 6-gingerol, 8-gingerol, 10-gingerol, 8-shogaol and 6-shogaol [51], which exhibit antioxidant activity. These compounds, more than just lending ginger its distinctive taste, have been found to exhibit a spectrum of effects, ranging from potential cancer protection to anti-inflammatory and antioxidant actions [52]. Ginger and its active components inhibit many enzymes and cytokines involved in pro-inflammatory pathways, such as cyclooxygenase-2, cytokines IL-1 and IL-6 [53-55]. In addition, ginger inhibits 5-lipooxygenase and cyclooxygenase-1 activity [56]. One study of ginger demonstrated a significant reduction in inflammatory cytokines (IL-12, Tumor necrosis factor-α and Interleukin-1 β) and pro-inflammatory chemokines in lipopolysaccharide-induced macrophages [57].

Other studies have demonstrated anti-inflammatory benefits of specific components of ginger. 6-Gingerol has been shown to inhibit Ikβα phosphorylation, nuclear factor kappa-β nuclear activation and protein kinase C-α translocation, which in turn inhibits calcium mobilization and disruption of mitochondrial membrane potential in lipopolysaccharide-stimulated macrophages, thereby inhibiting inducible nitric oxide synthase and tumor necrosis factor-α expression [58]. 10-gingerol, 8-shogaol, and 10-shogaol have been shown to inhibit cyclooxygenase-2, thereby significantly reducing inflammation [59]. Moreover, 8-shoagol showed significant inhibitory effects against TNFα-, IL-1β-, and IL-17-mediated inflammation and migration in both an RA patient and a 3D synovial culture system [60]. Another study demonstrated that 6-shogaol has barrier-protective effects, as it affects tumor necrosis factor-α-induced claudin-2 upregulation and claudin-1 disassembly via inhibition of phoshatidylinositol-3-kinase/Akt and nuclear factor kappa light chain enhancer of activated B-cell signalling [61].

Lastly a systemic review demonstrated that supplementation with ginger powder was associated with a significant reduction in DAS-28, high-sensitivity C-reactive protein, and IL-1β in RA patients [62, 63].

### Clinical Trials:

One randomized double-blind placebo-controlled trial assigned patients with active RA to either 1500 mg ginger powder daily or placebo. A statistically significant decrease in retinoic-acid-receptor-related orphan nuclear receptor gamma (RORγt) and T-bet gene expression and a significant increase in forkhead box P3 (FoxP3) gene expression were observed. This study sheds light on the possible mechanisms by which ginger may affect RA disease activity, namely through the improvement in immune system function by decreasing factors involved in inflammation and autoimmunity and by increasing FoxP3, a factor involved in tolerance [62].

### Recommended dose:

The optimal dosage of ginger as a supplement for RA management remains an area warranting further research. Studies have utilized between 750mg to 2000mg of ginger powder daily, divided into 2 or 3 doses. Absorption may be enhanced by taking ginger with food [64]. Further research is needed to provide definitive dosage recommendations. However, current evidence supports 1000-1500mg daily as an effective and well-tolerated dosage range for ginger's anti-inflammatory effects in RA management.

## Omega 3 fatty acids

Omega 3 fatty acids are essential nutrients that the human body cannot produce on its own, necessitating their intake through dietary sources. Fatty fish, such as salmon, mackerel, sardines, and trout, stand out as rich sources of eicosapentaenoic acid (EPA) and docosahexaenoic acid (DHA) - the two primary omega-3 fatty acids with therapeutic potential. Flaxseeds, chia seeds, and walnuts provide alpha-linolenic acid (ALA), a precursor that the body can convert into EPA and DHA, although the conversion rate is relatively low [65] (Table 6).

**Table 6:** Animal studies and clinical trials on Omega 3 fatty acids.

First Author	Year & Country	Dosage Used	Participants	Measured Factor	Results
Kremer et al.^74^	Various, USA	Various dosages	Varied adult human participants	Effect of marine n-3 PUFAs on rheumatoid arthritis	Varied results, generally showing benefit
Rajaei et al. (71)	2016, Iran	2 capsules daily (1.8g EPA, 2.1g DHA)	60 (49 female, 11 male), Mean age 42.4 years	Effect of Omega-3 on rheumatoid arthritis symptoms and drug consumption	Significant improvement in clinical measures, reduced need for analgesics, no weight changes
Piet Geusens (73).	1994, Belgium	2.6 gm/day or 1.3 gm/day of Omega-3	90 patients with active RA, ages not specified	Long-term effects of Omega-3 in RA patients	Improvement in global evaluation and pain, especially with 2.6 gm/day

### Mechanism of action:

Omega-3 fatty acids, particularly EPA and DHA, have garnered significant attention for their potential therapeutic effects in RA. The mechanism of action involves their anti-inflammatory properties. Omega-3 fatty acids serve as precursors to anti- inflammatory lipid mediators, such as resolvins and protectins, which actively resolve inflammation and contribute to the resolution of inflammatory processes [66]. The anti-inflammatory effects of n-3 fatty acids are achieved through various mechanisms, such as changing the composition of fatty acids in cell membrane phospholipids, interfering with lipid rafts, preventing the activation of the pro-inflammatory nuclear factor kappa B, which reduces the expression of inflammatory genes, stimulating the anti-inflammatory transcription factor NR1C3 (also known as peroxisome proliferator activated receptor γ), and interacting with the G protein-coupled receptor GPR120 [67]. Additionally, omega-3 fatty acids compete with pro-inflammatory omega-6 fatty acids for incorporation into cell membranes, leading to the production of less inflammatory eicosanoid [68]. These effects collectively modulate immune cell function, reduce the production of inflammatory cytokines, and influence the balance of pro-inflammatory and anti-inflammatory signaling pathways [69].

### Clinical trials:

A systematic review published in 2012 evaluating the use of marine omega-3 polyunsaturated fatty acids (n-3 PUFAs) n-3 PUFAs (fatty acid supplements) in RA showed that almost all of the trials included demonstrated positive clinical outcomes associated with n-3 PUFAs. Frequently observed advantages encompass a decrease in the duration of morning stiffness, a lower count of tender or swollen joints, diminished joint pain, enhanced grip strength, lowered pain or disease activity (as evaluated by either physicians or patients), and a decreased usage of NSAIDs [70].

A 2016 double-blind RCT done by Rajaei et al. involving 60 RA patients treated with disease- modifying antirheumatic drugs (DMARDs) examined the impact of n-3 polyunsaturated fatty acid (PUFA) supplementation on disease activity and remission was examined. The group receiving n-3 PUFA supplementation exhibited noteworthy enhancements in early morning stiffness (EMS), pain severity, physician's assessment, swollen joint count, tender joint count, and physical function [71].

### Recommended dose:

Various fish oil formulations exhibit varying levels of EPA and DHA, typically ranging from 20 to 40 percent of the total EPA+DHA content for most supplement formulations. The remaining portion typically consists of other omega-3 fatty acids, monounsaturated fats, saturated fats, along with gelatin or glycerin. Consequently, a standard 1 g capsule typically contains between 200 and 400 mg of EPA and DHA [72]. The optimal dosing remains unclear. Geusens et al. randomly assigned RA patients to high-dose n-3 PUFAs (2.6g/day), low-dose n-3 PUFAs plus olive oil (1.3g/day n-3 + 3g olive oil), or olive oil placebo (6g/day) for 12 months. Only the high-dose n-3 group demonstrated significant improvements in patient and physician assessments of pain and proportions benefiting [73]. Kremer et al. compared effects of high-dose n-3 PUFAs (54 mg/kg EPA and 36 mg/kg DHA), low-dose n-3 PUFAs (27 mg/kg EPA and 18 mg/kg DHA), and olive oil as placebo in RA patients over 30 weeks. Both n-3 doses improved joint counts and pain versus baseline. Anti-inflammatory effects were seen with all interventions [74]. More research is still needed to clarify optimal n-3 dosing and formulations for RA treatment.

## Willow bark

Willow bark is derived from the bark of various willow tree species and has a long history of traditional use for its medicinal properties. In recent times, it has gained popularity as a natural supplement with potential health benefits. The key active compound in willow bark is salicin, which has similarities to acetylsalicylic acid, the active component in aspirin. This has led to the exploration of willow bark as a botanical alternative for addressing various health concerns. The therapeutic effects of willow bark primarily stem from salicin's role as a precursor to salicylic acid. Once ingested, salicin is metabolized in the body to salicylic acid, which exhibits anti-inflammatory, analgesic, and antipyretic properties [75] (Table 7).

**Table 7:** The clinical trial on willow bark.

First Author	Year and Country	Dose	Participants	Measured Factor	Results
C. Biegert (79)	2004 Germany	240mg/day salicin from willow bark extract	Osteoarthritis and rheumatoid arthritis patients	Patient assessment of pain (VAS, primary outcome), Number of tender and swollen joints, HAQ disability index, Patient/physician global assessment, Quality of life (SF-36), Inflammatory markers (ESR, CRP)	No significant efficacy of willow bark extract in osteoarthritis or rheumatoid arthritis

### Mechanism of Action:

Various preclinical studies in animal models and cell cultures have explored potential mechanisms to explain the anti-inflammatory effects of willow bark extract. Specifically, experiments found that compounds in willow bark are able to inhibit several key drivers of inflammation, including TNFα) and COX-2. These compounds also inhibit transport of the nuclear factor-kB (NF- kB) transcription factor into the nucleus of activated immune cells including monocytes. By suppressing TNFα, COX-2, and NF-kB nuclear migration, willow bark interferes with inflammation at the molecular level in these cells [76]. A study by Ishikado et al. revealed a role for antioxidant pathways in willow bark's effects, different bioactive components beyond salicin, and specific molecular signaling cascades such as p38 that may underlie its antioxidant and anti-inflammatory actions [77].

### Clinical trials:

A recent meta-analysis compiled and reviewed the evidence from randomized controlled trials evaluating the efficacy and safety of willow bark preparations where the included studies evaluated both OA and RA. The meta-analysis results showed that compared to placebo, willow bark significantly reduced pain and improved health status in arthritis patients across the studies analyzed. There was no significant difference found between willow bark extract and placebo groups in the overall risk of side effects. While promising, the certainty and quality of evidence is still limited due to potential bias, small sample sizes, and inconsistencies among the included willow bark trials [78].

### Recommended dose:

The dosing most used in studies was two tablets of Willow bark extract twice daily for six weeks, corresponding to a dose of 240 mg salicin/day [79].

**Figure 1.**
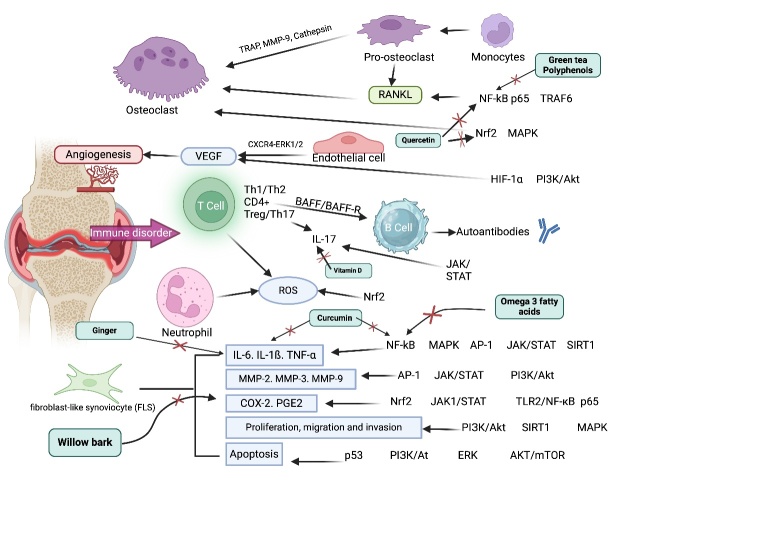
**This schematic diagram illustrates the complex pathophysiology of rheumatoid arthritis (RA) including a network of immune, molecular, and cellular interactions leading to chronic inflammation and joint destruction**. Central to this process are immune cells such as T cells, which facilitate the immune disorder, and B cells, which produce autoantibodies contributing to inflammation. Pro-inflammatory cytokines like IL-6, IL-1β, and TNF-α are released, which activate fibroblast-like synoviocytes and promote the secretion of matrix metalloproteinases. These degrade joint tissues. In the bloodstream, recruited to the inflamed joints and differentiate into osteoclasts under the influence of RANKL, leading to bone erosion. Concurrently, angiogenesis is stimulated by factors such as VEGF, nourishing the inflamed synovium. The oxidative stress marked by increased ROS further exacerbates tissue damage. The image also depicts the inhibitory effects of various natural reagents, such as vitamin D, curcumin, omega-3 fatty acids, green tea polyphenols, willow bark, and quercetin, which interfere with these pathogenic pathways. These compounds target pivotal molecular pathways, including NF-κB, IL-6, Nrf2, and others, indicating their potential in moderating the inflammatory and degenerative processes of RA.

## Discussion

The exploration of OTC anti-inflammatory supplements in the management of RA represents a significant adjunctive strategy to conventional pharmacotherapy. This review underscores the potential of various supplements including quercetin, green tea polyphenols, and omega-3 fatty acids in mitigating the inflammatory processes and symptomatology associated with RA. While conventional treatments like DMARDs, NSAIDs, and steroids remain the cornerstone of RA management, their associated side effects and limitations necessitate complementary approaches. The reviewed supplements, with their anti-inflammatory and immunomodulatory properties, offer promising alternatives.

**Table 8:** Summarizing mechanism of action, clinical trials, and recommendations.

Supplement	Mechanism of Action	Clinical Trials	Recommended Dosage
Quercetin	Reduces inflammatory cytokines; blocks signaling pathways	Demonstrated improvement in RA symptoms and inflammatory markers in women.	500-1000 mg/day
Green Tea Polyphenols	Inhibits inflammatory mediators; suppresses pathways	Positive outcomes in animal studies; limited human trials.	500-1000mg/day (standardized extract)
Vitamin D	Modulates T cell populations; suppresses proinflammatory cells	Mixed outcomes in RA studies, some showing improvement.	600-4000 IU/day
Curcumin	Inhibits inflammatory cytokines and pathways	Showed notable improvement over diclofenac sodium in RA treatment.	500 mg/day
Ginger	Inhibits enzymes, cytokines, and inflammation pathways	Significant reduction in inflammation markers in RA patients.	750mg-2000mg/day (ginger powder)
Omega-3 Fatty Acids	Precursors to anti-inflammatory mediators; reduces cytokines	Several studies showing positive clinical outcomes in RA.	Varies (e.g., 2.6g/day)
Willow Bark	Inhibits inflammation	Mixed results in RA, with some studies showing benefit.	240 mg salicin/day

The rising patient interest in OTC supplements is driven by the desire to minimize pharmaceutical side effects and enhance overall quality of life. However, this interest must be tempered with a cautionary approach due to the variability in supplement quality, potential side effects, and interactions with conventional RA medications.

While these supplements show promise due to their anti-inflammatory properties, several crucial concerns need addressing. Firstly, the inconsistency in the quality and formulation of these supplements is a significant issue, potentially impacting both safety and efficacy. This variability underscores the need for stringent quality control and standardization in the supplement industry. Moreover, the interaction of these supplements with conventional RA medications remains a grey area, posing risks of adverse reactions and potentially diminishing the effectiveness of established RA treatments. This lack of clarity necessitates cautious integration of these supplements into existing RA management protocols. Additionally, a major gap in the literature is the absence of established standard dosages for RA, leading to uncertainties in their therapeutic application. This highlights the need for clinical guidelines based on robust research to determine optimal dosages for RA patients.

While current studies have shed light on the efficacy of these supplements, there is an evident need for more extensive, methodologically sound randomized controlled trials. Such studies are crucial to conclusively determine the safety, effectiveness, and appropriate dosing of these supplements in RA treatment. Lastly, it is important to acknowledge the variability in individual responses to supplements, influenced by genetics, health conditions, and lifestyle factors. This variation suggests that the effectiveness of supplements may differ from one individual to another, emphasizing the need for personalized approaches in RA management.

In summary, this review highlights the potential role of OTC anti-inflammatory supplements as an adjunctive treatment in RA management. While supplements such as quercetin, green tea polyphenols, and omega-3 fatty Acids exhibit promising anti-inflammatory and immunomodulatory effects, their integration into clinical practice necessitates further rigorous research. The growing interest in these supplements underscores the need for a balanced approach that prioritizes patient safety and efficacy. Ultimately, the goal is to enhance RA treatment paradigms, offering patients complementary options that align with the principles of evidence-based medicine and holistic care.

## References

[1] Boersma P, Black LI, Ward BW (2018). Peer reviewed: Prevalence of multiple chronic conditions among US adults. Preventing chronic disease, 17. 10.5888/pcd17.200130 PMC7553211 32945769

[2] RaghupathiW, RaghupathiV (2018). An empirical study of chronic diseases in the United States: A visual analytics approach to public health. International journal of environmental research and public health, 15(3):431.29494555 10.3390/ijerph15030431PMC5876976

[3] HunterTM, BoytsovNN, ZhangX, SchroederK, MichaudK, AraujoAB (2017). Prevalence of rheumatoid arthritis in the United States adult population in healthcare claims databases, 2004-2014. Rheumatol Int, 37:1551-7.28455559 10.1007/s00296-017-3726-1

[4] KiadaliriAA, WoolfAD, EnglundM (2017). Musculoskeletal disorders as underlying cause of death in 58 countries, 1986-2011: Trend analysis of WHO mortality database. BMC musculoskeletal disorders, 18:1-12.28153007 10.1186/s12891-017-1428-1PMC5290674

[5] RaychaudhuriS, SandorC, StahlEA, FreudenbergJ, LeeH, JiaX, et al. (2012). Five amino acids in three HLA proteins explain most of the association between MHC and seropositive rheumatoid arthritis. Nat Genet, 44(3):291-6.22286218 10.1038/ng.1076PMC3288335

[6] Smolen JS (2020). Rheumatoid arthritis primer—behind the scenes. Nature Reviews Disease Primers, 6(1):32. 10.1038/s41572-020-0168-y 32327647

[7] RaduA, BungauSG (2021). Management of rheumatoid arthritis: An overview. Cells, 10(11):2857.34831081 10.3390/cells10112857PMC8616326

[8] Grigor C, Capell H, Stirling A, McMahon AD, Lock P, Vallance R, et al. (2004). Effect of a treatment strategy of tight control for rheumatoid arthritis (the TICORA study): A single-blind randomised controlled trial. The Lancet, 364(9430):263-9. 10.1016/S0140-6736(04)16676-2 15262104

[9] MuellerA, PayandehZ, MohammadkhaniN, MubarakSM, ZakeriA, Alagheband BahramiA, et al. (2021). Recent advances in understanding the pathogenesis of rheumatoid arthritis: New treatment strategies. Cells, 10(11):3017.34831240 10.3390/cells10113017PMC8616543

[10] Lindstrom A, Ooyen C, Lynch ME, Blumenthal M (2013). Herb supplement sales increase 5.5% in 2012: Herbal supplement sales rise for 9th consecutive year; turmeric sales jump 40% in natural channel. HerbalGram, 99:60-5.

[11] SharmaD, ChaubeyP, SuvarnaV (2021). Role of natural products in alleviation of rheumatoid arthritis—A review. J Food Biochem, 45(4):e13673.33624882 10.1111/jfbc.13673

[12] Boisset M, Fitzcharles M (1994). Alternative medicine use by rheumatology patients in a universal health care setting. J Rheumatol, 21(1):148-52. 8151571

[13] Falci L, Shi Z, Greenlee H (2016). Peer reviewed: Multiple chronic conditions and use of complementary and alternative medicine among US adults: Results from the 2012 national health interview survey. Preventing chronic disease, 13 10.5888/pcd13.150501 PMC4858448 27149072

[14] Singh JA, Saag KG, Bridges SL Jr, Akl EA, Bannuru RR, Sullivan MC, et al. (2016). American college of rheumatology guideline for the treatment of rheumatoid arthritis. Arthritis & rheumatology, 68(1):1-26. 10.1002/art.39480 26545940

[15] SkibaMB, HopkinsLL, HopkinsAL, BillheimerD, FunkJL (2020). Nonvitamin, nonmineral dietary supplement use in individuals with rheumatoid arthritis. J Nutr, 150(9):2451-9.32805045 10.1093/jn/nxaa197PMC7540062

[16] Nattagh-EshtivaniE, PahlavaniN, RanjbarG, Gholizadeh NavashenaqJ, Salehi-SahlabadiA, MahmudionoT, et al. (2022). Does propolis have any effect on rheumatoid arthritis? A review study. Food science & nutrition, 10(4):1003-20.35432965 10.1002/fsn3.2684PMC9007309

[17] Nattagh-EshtivaniE, GheflatiA, BarghchiH, RahbarinejadP, HachemK, ShalabyMN, et al. (2022). The role of pycnogenol in the control of inflammation and oxidative stress in chronic diseases: Molecular aspects. Phytotherapy Research, 36(6):2352-74.35583807 10.1002/ptr.7454

[18] PuteraHD, DoewesRI, ShalabyMN, Ramírez-CoronelAA, ClaytonZS, AbdelbassetWK, et al. (2023). The effect of conjugated linoleic acids on inflammation, oxidative stress, body composition and physical performance: A comprehensive review of putative molecular mechanisms. Nutrition & Metabolism, 20(1):35.37644566 10.1186/s12986-023-00758-9PMC10466845

[19] FiresteinGS, McInnesIB (2017). Immunopathogenesis of rheumatoid arthritis. Immunity, 46(2):183-96.28228278 10.1016/j.immuni.2017.02.006PMC5385708

[20] GuardiaT, RotelliAE, JuarezAO, PelzerLE (2001). Anti-inflammatory properties of plant flavonoids. effects of rutin, quercetin and hesperidin on adjuvant arthritis in rat. Il farmaco, 56(9):683-7.11680812 10.1016/s0014-827x(01)01111-9

[21] YuanK, ZhuQ, LuQ, JiangH, ZhuM, LiX, et al. (2020). Quercetin alleviates rheumatoid arthritis by inhibiting neutrophil inflammatory activities. J Nutr Biochem, 84:108454.32679549 10.1016/j.jnutbio.2020.108454

[22] ChoiE, BaeS, YuR, YounJ, SungM (2009). Dietary vitamin E and quercetin modulate inflammatory responses of collagen-induced arthritis in mice. Journal of medicinal food, 12(4):770-5.19735175 10.1089/jmf.2008.1246

[23] JavadiF, AhmadzadehA, EghtesadiS, AryaeianN, ZabihiyeganehM, Rahimi ForoushaniA, et al. (2017). The effect of quercetin on inflammatory factors and clinical symptoms in women with rheumatoid arthritis: A double-blind, randomized controlled trial. J Am Coll Nutr, 36(1):9-15.27710596 10.1080/07315724.2016.1140093

[24] EgertS, WolfframS, Bosy-WestphalA, Boesch-SaadatmandiC, WagnerAE, FrankJ, et al. (2008). Daily quercetin supplementation dose-dependently increases plasma quercetin concentrations in healthy humans. J Nutr, 138(9):1615-21.18716159 10.1093/jn/138.9.1615

[25] SinghR, AhmedS, MalemudCJ, GoldbergVM, HaqqiTM (2003). Epigallocatechin-3-gallate selectively inhibits interleukin-1 β-induced activation of mitogen activated protein kinase subgroup c-Jun n-terminal kinase in human osteoarthritis chondrocytes. Journal of orthopaedic research, 21(1):102-9.12507586 10.1016/S0736-0266(02)00089-X

[26] Ahmed S (2010). Green tea polyphenol epigallocatechin 3-gallate in arthritis: Progress and promise. Arthritis research & therapy, 12:1-9. 10.1186/ar2982 PMC2888220 20447316

[27] ForesterSC, LambertJD (2011). The role of antioxidant versus pro-oxidant effects of green tea polyphenols in cancer prevention. Molecular nutrition & food research, 55(6):844-54.21538850 10.1002/mnfr.201000641PMC3679539

[28] Haqqi TM, Anthony DD, Gupta S, Ahmad N, Lee M, Kumar GK, et al. (1999). Prevention of collagen-induced arthritis in mice by a polyphenolic fraction from green tea. Proceedings of the National Academy of Sciences, 96(8):4524-9. 10.1073/pnas.96.8.4524 PMC16365 10200295

[29] Lin S, Chang H, Chen Y, Wang C, Galson DL, Hong C, et al. (2008). Epigallocatechin-3-gallate diminishes CCL2 expression in human osteoblastic cells via up-regulation of phosphatidylinositol 3-kinase/akt/Raf-1 interaction: A potential therapeutic benefit for arthritis. Arthritis & Rheumatism: Official Journal of the American College of Rheumatology, 58(10):3145-56. 10.1002/art.23937 18821707

[30] Morinobu A, Biao W, Tanaka S, Horiuchi M, Jun L, Tsuji G, et al. (2008). Epigallocatechin-3-gallate suppresses osteoclast differentiation and ameliorates experimental arthritis in mice. Arthritis & Rheumatism: Official Journal of the American College of Rheumatology, 58(7):2012-8. 10.1002/art.23594 18576345

[31] IsbruckerRA, BauschJ, EdwardsJA, WolzE (2006). Safety studies on epigallocatechin gallate (EGCG) preparations. part 1: Genotoxicity. Food and Chemical Toxicology, 44(5):626-35.16364532 10.1016/j.fct.2005.07.005

[32] ArnsonY, AmitalH, ShoenfeldY (2007). Vitamin D and autoimmunity: New aetiological and therapeutic considerations. Ann Rheum Dis, 66(9):1137-42.17557889 10.1136/ard.2007.069831PMC1955167

[33] GuanY, HaoY, GuanY, BuH, WangH (2020). The effect of vitamin D supplementation on rheumatoid arthritis patients: A systematic review and meta-analysis. Frontiers in Medicine, 7:596007.33195358 10.3389/fmed.2020.596007PMC7661491

[34] ChangSH, ChungY, DongC (2010). Vitamin D suppresses Th17 cytokine production by inducing C/EBP homologous protein (CHOP) expression. J Biol Chem, 285(50):38751-5.20974859 10.1074/jbc.C110.185777PMC2998156

[35] SzélesL, KeresztesG, TöröcsikD, BalajthyZ, KrenácsL, PóliskaS, et al. (2009). 1, 25-dihydroxyvitamin D3 is an autonomous regulator of the transcriptional changes leading to a tolerogenic dendritic cell phenotype. The Journal of Immunology, 182(4):2074-83.19201860 10.4049/jimmunol.0803345

[36] DehghanA, RahimpourS, Soleymani-SalehabadiH, OwliaMB (2014). Rolle von vitamin D bei schüben der rheumatoiden arthritis. Zeitschrift für Rheumatologie, 73:461-4.24352479 10.1007/s00393-013-1297-4

[37] ChandrashekaraS, PattedA (2017). Role of vitamin D supplementation in improving disease activity in rheumatoid arthritis: An exploratory study. International journal of rheumatic diseases, 20(7):825-31.26481198 10.1111/1756-185X.12770

[38] BuondonnoI, RoveraG, SassiF, RigoniMM, LomaterC, ParisiS, et al. (2017). Vitamin D and immunomodulation in early rheumatoid arthritis: A randomized double-blind placebo-controlled study. PLoS One, 12(6):e0178463.28582403 10.1371/journal.pone.0178463PMC5459341

[39] GopinathK, DandaD (2011). Supplementation of 1, 25 dihydroxy vitamin D3 in patients with treatment naive early rheumatoid arthritis: A randomised controlled trial. International journal of rheumatic diseases, 14(4):332-9.22004229 10.1111/j.1756-185X.2011.01684.x

[40] Franco AS, Freitas TQ, Bernardo WM, Pereira RMR (2017). Vitamin D supplementation and disease activity in patients with immune-mediated rheumatic diseases: A systematic review and meta-analysis. Medicine, 96(23) 10.1097/MD.0000000000007024 PMC5466211 28591033

[41] PrietlB, TreiberG, PieberTR, AmreinK (2013). Vitamin D and immune function. Nutrients, 5(7):2502-21.23857223 10.3390/nu5072502PMC3738984

[42] HolickMF, BinkleyNC, Bischoff-FerrariHA, GordonCM, HanleyDA, HeaneyRP, et al. (2011). Evaluation, treatment, and prevention of vitamin D deficiency: An endocrine society clinical practice guideline. The Journal of clinical endocrinology & metabolism, 96(7):1911-30.21646368 10.1210/jc.2011-0385

[43] Prasad S, Aggarwal BB (2011). Turmeric, the golden spice. Herbal Medicine: Biomolecular and Clinical Aspects. 2nd edition.

[44] FuY, GaoR, CaoY, GuoM, WeiZ, ZhouE, et al. (2014). Curcumin attenuates inflammatory responses by suppressing TLR4-mediated NF-κB signaling pathway in lipopolysaccharide-induced mastitis in mice. Int Immunopharmacol, 20(1):54-8.24508537 10.1016/j.intimp.2014.01.024

[45] ChandranB, GoelA (2012). A randomized, pilot study to assess the efficacy and safety of curcumin in patients with active rheumatoid arthritis. Phytotherapy research, 26(11):1719-25.22407780 10.1002/ptr.4639

[46] Pourhabibi-ZarandiF, RafrafM, ZayeniH, Asghari-JafarabadiM, EbrahimiA (2022). Effects of curcumin supplementation on metabolic parameters, inflammatory factors and obesity values in women with rheumatoid arthritis: A randomized, double-blind, placebo-controlled clinical trial. Phytotherapy Research, 36(4):1797-806.35178811 10.1002/ptr.7422

[47] JavadiM, Khadem HaghighianH, GoodarzyS, AbbasiM, Nassiri-AslM (2019). Effect of curcumin nanomicelle on the clinical symptoms of patients with rheumatoid arthritis: A randomized, double-blind, controlled trial. International journal of rheumatic diseases, 22(10):1857-62.31482684 10.1111/1756-185X.13688

[48] Hsieh C (2001). Phase I clinical trial of curcumin, a chemopreventive agent, in patients with high-risk or pre-malignant lesions. Anticancer Res, 21(2895):e2900. 11712783

[49] Liu Y, Liu J, Zhang Y (2019). Research progress on chemical constituents of zingiber officinale roscoe. BioMed research international. 10.1155/2019/5370823 PMC6942719 31930125

[50] CrichtonM, DavidsonAR, InnerarityC, MarxW, LohningA, IsenringE, et al. (2022). Orally consumed ginger and human health: An umbrella review. Am J Clin Nutr, 115(6):1511-27.35147170 10.1093/ajcn/nqac035PMC9170469

[51] MaoQ, XuX, CaoS, GanR, CorkeH, BetaT, et al. (2019). Bioactive compounds and bioactivities of ginger (zingiber officinale roscoe). Foods, 8(6):185.31151279 10.3390/foods8060185PMC6616534

[52] de LimaRMT, Dos ReisAC, de MenezesAPM, SantosJVdO, FilhoJWGdO, FerreiraJRdO, et al. (2018). Protective and therapeutic potential of ginger (zingiber officinale) extract and [6]-gingerol in cancer: A comprehensive review. Phytotherapy research, 32(10):1885-907.30009484 10.1002/ptr.6134

[53] KiuchiF, IwakamiS, ShibuyaM, HanaokaF, SankawaU (1992). Inhibition of prostaglandin and leukotriene biosynthesis by gingerols and diarylheptanoids. Chemical and Pharmaceutical bulletin, 40(2):387-91.1606634 10.1248/cpb.40.387

[54] Ribel-Madsen S, Bartels EM, Stockmarr A, Borgwardt A, Cornett C, Danneskiold-Samsøe B, et al. (2012). A synoviocyte model for osteoarthritis and rheumatoid arthritis: Response to ibuprofen, betamethasone, and ginger extract—a cross-sectional in vitro study. Arthritis. 10.1155/2012/505842 PMC3546442 23365744

[55] SrivastavaKC, MustafaT (1992). Ginger (zingiber officinale) in rheumatism and musculoskeletal disorders. Med Hypotheses, 39(4):342-8.1494322 10.1016/0306-9877(92)90059-l

[56] Nurtjahja-TjendraputraE, AmmitAJ, RoufogalisBD, TranVH, DukeCC (2003). Effective anti-platelet and COX-1 enzyme inhibitors from pungent constituents of ginger. Thromb Res, 111(4-5):259-65.14693173 10.1016/j.thromres.2003.09.009

[57] TripathiS, BruchD, KitturDS (2008). Ginger extract inhibits LPS induced macrophage activation and function. BMC complementary and Alternative Medicine, 8:1-7.18173849 10.1186/1472-6882-8-1PMC2234390

[58] LeeT, LeeK, ChenS, ChangH (2009). 6-gingerol inhibits ROS and iNOS through the suppression of PKC-α and NF-κB pathways in lipopolysaccharide-stimulated mouse macrophages. Biochem Biophys Res Commun, 382(1):134-9.19268427 10.1016/j.bbrc.2009.02.160

[59] van BreemenRB, TaoY, LiW (2011). Cyclooxygenase-2 inhibitors in ginger (zingiber officinale). Fitoterapia, 82(1):38-43.20837112 10.1016/j.fitote.2010.09.004PMC3018740

[60] JoS, SamarpitaS, LeeJS, LeeYJ, SonJE, JeongM, et al. (2022). 8-shogaol inhibits rheumatoid arthritis through targeting TAK1. Pharmacological Research, 178:106176.35283302 10.1016/j.phrs.2022.106176

[61] LuettigJ, RosenthalR, LeeIM, KrugSM, SchulzkeJD (2016). The ginger component 6-shogaol prevents TNF-α-induced barrier loss via inhibition of PI3K/akt and NF-κB signaling. Molecular nutrition & food research, 60(12):2576-86.27487982 10.1002/mnfr.201600274

[62] Aryaeian N, Mahmoudi M, Shahram F, Poursani S, Jamshidi F, Tavakoli H (2019). The effect of ginger supplementation on IL2, TNFα, and IL1β cytokines gene expression levels in patients with active rheumatoid arthritis: A randomized controlled trial. Med J Islamic Rep Iran, 33:154. 10.34171/mjiri.33.154 PMC7137811 32280660

[63] LongZ, XiangW, HeQ, XiaoW, WeiH, LiH, et al. (2023). Efficacy and safety of dietary polyphenols in rheumatoid arthritis: A systematic review and meta-analysis of 47 randomized controlled trials. Frontiers in Immunology, 14:1024120.37033930 10.3389/fimmu.2023.1024120PMC10073448

[64] MukkavilliR, YangC, Singh TanwarR, GhareebA, LuthraL, AnejaR (2017). Absorption, metabolic stability, and pharmacokinetics of ginger phytochemicals. Molecules, 22(4):553.28358331 10.3390/molecules22040553PMC6154694

[65] Calder P (2017). Omega-3: The good oil. Nutr Bull, 42(2):132-40.

[66] CalderPC (2015). Marine omega-3 fatty acids and inflammatory processes: Effects, mechanisms and clinical relevance. Biochimica et Biophysica Acta (BBA)-Molecular and Cell Biology of Lipids, 1851(4):469-84.25149823 10.1016/j.bbalip.2014.08.010

[67] CalderPC (2013). Omega-3 polyunsaturated fatty acids and inflammatory processes: Nutrition or pharmacology? Br J Clin Pharmacol, 75(3):645-62.22765297 10.1111/j.1365-2125.2012.04374.xPMC3575932

[68] WongSW, KwonM, ChoiAM, KimH, NakahiraK, HwangDH (2009). Fatty acids modulate toll-like receptor 4 activation through regulation of receptor dimerization and recruitment into lipid rafts in a reactive oxygen species-dependent manner. J Biol Chem, 284(40):27384-92.19648648 10.1074/jbc.M109.044065PMC2785667

[69] CaugheyGE, MantziorisE, GibsonRA, ClelandLG, JamesMJ (1996). The effect on human tumor necrosis factor alpha and interleukin 1 beta production of diets enriched in n-3 fatty acids from vegetable oil or fish oil. Am J Clin Nutr, 63(1):116-22.8604658 10.1093/ajcn/63.1.116

[70] MilesEA, CalderPC (2012). Influence of marine n-3 polyunsaturated fatty acids on immune function and a systematic review of their effects on clinical outcomes in rheumatoid arthritis. Br J Nutr, 107(S2):S171-84.22591891 10.1017/S0007114512001560

[71] Rajaei E, Mowla K, Ghorbani A, Bahadoram S, Bahadoram M, Dargahi-Malamir M (2016). The effect of omega-3 fatty acids in patients with active rheumatoid arthritis receiving DMARDs therapy: Double-blind randomized controlled trial. Global journal of health science, 8(7):18. 10.5539/gjhs.v8n7p18 PMC4965662 26925896

[72] CheeKM, GongJX, Good ReesDM, MeydanlM, AusmanL, JohnsonJ, et al. (1990). Fatty acid content of marine oil capsules. Lipids, 25:523-8.2250588 10.1007/BF02537158

[73] Geusens P, Wouters C, Nijs J, Jiang Y, Dequeker J (1994). Long-term effect of omega-3 fatty acid supplementation in active rheumatoid arthritis. Arthritis & Rheumatism: Official Journal of the American College of Rheumatology, 37(6):824-9. 10.1002/art.1780370608 8003055

[74] Kremer JM, Lawrence DA, Jubiz W, Digiacomo R, Rynes R, Bartholomew LE, et al. (1990). Dietary fish oil and olive oil supplementation in patients with rheumatoid arthritis clinical and immunologic effects. Arthritis & Rheumatism: Official Journal of the American College of Rheumatology, 33(6):810-20. 10.1002/art.1780330607 2363736

[75] Kammerer B, Kahlich R, Biegert C, Gleiter CH, Heide L (2005). HPLC-MS/MS analysis of willow bark extracts contained in pharmaceutical preparations. Phytochemical Analysis: An International Journal of Plant Chemical and Biochemical Techniques, 16(6):470-8. 10.1002/pca.873 16315493

[76] BonaterraGA, HeinrichEU, KelberO, WeiserD, MetzJ, KinscherfR (2010). Anti-inflammatory effects of the willow bark extract STW 33-I (proaktiv®) in LPS-activated human monocytes and differentiated macrophages. Phytomedicine, 17(14):1106-13.20570123 10.1016/j.phymed.2010.03.022

[77] IshikadoA, SonoY, MatsumotoM, Robida-StubbsS, OkunoA, GotoM, et al. (2013). Willow bark extract increases antioxidant enzymes and reduces oxidative stress through activation of Nrf2 in vascular endothelial cells and caenorhabditis elegans. Free Radical Biology and Medicine, 65:1506-15.23277146 10.1016/j.freeradbiomed.2012.12.006PMC3800243

[78] LinC, TsaiSHL, WangC, LeeC, HungS, TingY, et al. (2023). Willow bark (salix spp.) used for pain relief in arthritis: A meta-analysis of randomized controlled trials. Life, 13(10):2058.37895439 10.3390/life13102058PMC10607963

[79] Biegert C, Wagner I, Lüdtke R, Kötter I, Lohmüller C, Günaydin I, et al. (2004). Efficacy and safety of willow bark extract in the treatment of osteoarthritis and rheumatoid arthritis: Results of 2 randomized double-blind controlled trials. J Rheumatol, 31(11):2121-30. 15517622

